# Likelihood of infection in patients with presumed sepsis at the time of intensive care unit admission: a cohort study

**DOI:** 10.1186/s13054-015-1035-1

**Published:** 2015-09-07

**Authors:** Peter M. C. Klein Klouwenberg, Olaf L. Cremer, Lonneke A. van Vught, David S. Y. Ong, Jos F. Frencken, Marcus J. Schultz, Marc J. Bonten, Tom van der Poll

**Affiliations:** Department of Intensive Care Medicine, University Medical Center Utrecht, Room F06.149, P.O. Box 85500, 3508 GA Utrecht, The Netherlands; Department of Medical Microbiology, University Medical Center Utrecht, P.O. Box 85500, 3508 GA Utrecht, The Netherlands; Julius Center for Health Sciences and Primary Care, University Medical Center Utrecht, P.O. Box 85500, 3508 GA Utrecht, The Netherlands; Center for Experimental and Molecular Medicine and Division of Infectious Diseases, Academic Medical Center, University of Amsterdam, Meibergdreef 9, 1105 AZ Amsterdam, The Netherlands; Department of Intensive Care Medicine, Academic Medical Center, University of Amsterdam, Meibergdreef 9, 1105 AZ Amsterdam, The Netherlands

## Abstract

**Introduction:**

A clinical suspicion of infection is mandatory for diagnosing sepsis in patients with a systemic inflammatory response syndrome. Yet, the accuracy of categorizing critically ill patients presenting to the intensive care unit (ICU) as being infected or not is unknown. We therefore assessed the likelihood of infection in patients who were treated for sepsis upon admission to the ICU, and quantified the association between plausibility of infection and mortality.

**Methods:**

We studied a cohort of critically ill patients admitted with clinically suspected sepsis to two tertiary ICUs in the Netherlands between January 2011 and December 2013. The likelihood of infection was categorized as none, possible, probable or definite by post-hoc assessment. We used multivariable competing risks survival analyses to determine the association of the plausibility of infection with mortality.

**Results:**

Among 2579 patients treated for sepsis, 13% had a post-hoc infection likelihood of “none”, and an additional 30% of only “possible”. These percentages were largely similar for different suspected sites of infection. In crude analyses, the likelihood of infection was associated with increased length of stay and complications. In multivariable analysis, patients with an unlikely infection had a higher mortality rate compared to patients with a definite infection (subdistribution hazard ratio 1.23; 95% confidence interval 1.03-1.49).

**Conclusions:**

This study is the first prospective analysis to show that the clinical diagnosis of sepsis upon ICU admission corresponds poorly with the presence of infection on post-hoc assessment. A higher likelihood of infection does not adversely influence outcome in this population.

**Trial registration:**

ClinicalTrials.gov NCT01905033. Registered 11 July 2013.

**Electronic supplementary material:**

The online version of this article (doi:10.1186/s13054-015-1035-1) contains supplementary material, which is available to authorized users.

## Introduction

Sepsis is a syndrome that arises when the body’s response to a severe infection injures its own tissues. In 1992 an international consensus panel proposed a clinical definition for sepsis, making use of the concept of a “systemic inflammatory response syndrome” (SIRS), involving alterations in body temperature, heart rate, respiration rate, and leukocyte counts [1]. The panel defined sepsis as SIRS caused by suspected infection. It further introduced the terms severe sepsis to describe cases when sepsis is complicated by acute organ dysfunction and septic shock as severe sepsis complicated by hypotension refractory to fluid resuscitation. These definitions, generally referred to as the “Bone criteria”, have been used as inclusion criteria in many clinical sepsis trials, and until today have remained largely unchanged [2].

Although the clinical suspicion of infection is a crucial factor in making a sepsis diagnosis, little is known about the accuracy of this diagnosis in the context of critically ill patients who present to the ICU with signs and symptoms of a “sepsis syndrome”. We hypothesized that in the clinical practice of an ICU the diagnosis of sepsis is not based on strict diagnostic criteria for infection and that as a consequence the occurrence of sepsis on the ICU might be overestimated. Quantification of this discordance is helpful for estimating incidence rates in epidemiological studies and the possible reduction of antibiotic use.

To address this hypothesis we assessed the concordance between the prospective clinical sepsis diagnosis made by bedside physicians and the post-hoc diagnosis of infection made by clinical researchers using strict criteria. In addition, we assessed the association of the likelihood of infection with outcome.

## Methods

### Study design and population

This cohort study was incorporated in the Molecular Diagnosis and Risk Stratification of Sepsis (MARS) project in the mixed ICUs of two tertiary referral centers in the Netherlands [3, 4]. The local ethical committee approved the study with opt-out consent (Medisch Ethische Toetsingscommissie UMC Utrecht; number 10-056C; approval 16 June 2010). Participants were notified of the study in writing by a brochure provided at ICU admission with an attached opt-out card that could be completed by the patient or by his or her legal representative in case of unwillingness to participate. For the current study, we analyzed all first admissions of adult patients with a sepsis diagnosis who were admitted between January 2011 and December 2013, with an expected stay of >24 hours.

### Data collection and definitions

Dedicated and trained observers prospectively collected relevant data from all patients. An infectious event was prospectively recorded when systemic antibiotics were started for therapeutic reasons by the attending physician, as described previously [3]. For this study, we included infections that were diagnosed before ICU admission or until 48 hours afterwards. Patients who were initially not suspected of having an infection at admission (i.e., those not receiving therapeutic antibiotics at ICU admission), but in whom therapeutic antibiotics were started later than 2 days after admittance because the positive cultures and the continuous presence of clinical symptoms from the time of admission onwards suggested an infection in retrospect, were also included in this study. The clinical research team in these cases dated the sepsis event to the start of clinical symptoms. We performed a sensitivity analysis in which we excluded infections starting more than 48 hours before admission, because we anticipated that the diagnostic likelihood of these infections differed from those starting directly prior to admission. Of note, patients receiving (only) prophylactic antibiotics were not assumed to have sepsis.

The plausibility of infection (none, possible, probable, definite) was determined post hoc, based on all available clinical, microbiological, and radiological evidence and according to the Centers for Disease Control and Prevention (CDC) and the International Sepsis Forum (ISF) criteria [5, 6]. This post-hoc assessment was used as the “gold standard” for infection in our study. In short, independent observers discussed all patients with (senior) critical care physicians and infection specialists in daily multidisciplinary meetings, and performed a confirmatory review of the medical record at the time of ICU discharge or death, including any postmortem findings. All diagnoses were therefore made after consensus and continuous data integrity checks. The precise definitions used to diagnose infection in the present study are described in a previous publication by our consortium [3].

SIRS criteria were defined as: temperature <36.0 °C or >38.0 °C during at least 2 hours and 1 hour, respectively; white blood cell count <4 × 10^9^/l or >12 × 10^9^/l, or >10 % immature (band) forms; heart rate >90/minute during at least 1 hour; respiratory rate >20/minute during at least 1 hour, partial pressure of carbon dioxide <32 mmHg, or mechanical ventilation [1, 7]. Sepsis was defined as at least two SIRS criteria plus the clinical suspicion of infection by senior clinicians; we chose this “conventional” sepsis definition making use of the SIRS criteria to allow for interpretation in the context of the many previous sepsis trials using these criteria [2]. In addition, patients with “do not resuscitate” orders with clinical suspicion of sepsis, but without antibiotics, were classified as having sepsis. Criteria for organ failure included the following signs of organ hypoperfusion or dysfunction: areas of mottled skin; capillary refilling requiring 3 seconds or longer; urine output <0.5 ml/kg for at least 6 hours, elevated creatinine, or renal replacement therapy [8]; lactate >2 mmol/l; abrupt change in mental status; abnormal electroencephalographic findings; platelet count <100,000 platelets/ml or disseminated intravascular coagulation; acute respiratory distress syndrome; and cardiac dysfunction, as defined by echocardiography or direct measurement of the cardiac index [9]. Shock was defined as the presence of severe sepsis plus the use of noradrenaline at a dose of >0.1 μg/kg/minute during at least 50 % of the day. ICU-related complications such as ICU-acquired infections, acute kidney injury, and adult respiratory distress syndrome that were present at or occurred during ICU admission were prospectively registered [10, 11]. For ICU-acquired infections, only infections that started >48 hours after admission with a probability of at least “possible” were included.

All patients with sepsis were managed according to locally adapted protocols based on the Surviving Sepsis Campaign guidelines [12].

### Statistical analyses

All results are presented as median and interquartile range (IQR) or number and percentage, as appropriate. Continuous nonparametric data were analyzed using a Kruskal–Wallis test and categorical data were analyzed using the chi-squared test. The Cochran–Armitage test for trend and the Gray’s test for equality of cumulative incidence functions were used.

We assessed the effect of infection plausibility on ICU mortality using a competing risks survival analysis to account for informative censoring [13]. A competing risks analysis provides two measures of association: the cause-specific hazard ratio (CSHR), which estimates the direct effects of infection on outcome (both ICU discharge and death); and the subdistribution hazard ratio (SHR), which is a summary measure of all separate cause-specific hazards and can be used to calculate the cumulative incidence of the outcome of interest (i.e., death in this study) [14]. The plausibility of infection was included as a dichotomous variable (none/possible vs. probable/definite). We adjusted for confounders that were chosen a priori based on their expected associations with infection and mortality after careful consideration of the literature and based on clinical expertise. These included age, gender, cardiovascular disease, immunocompromised state, malignancy, diabetes mellitus, respiratory insufficiency, renal insufficiency, recent surgery, sepsis severity, site of infection, and Acute Physiology and Chronic Health Evaluation (APACHE) IV score. *p* <0.05 was considered statistically significant. All analyses were performed using SAS 9.2 (SAS Institute, Cary, NC, USA) and R version 3.10 [15].

## Results

### Demographics

Over the 3-year observation period we studied 6944 patients during 7347 hospitalizations contributing a total of 8259 ICU episodes, of which 912 ICU readmissions were excluded from analysis. At admission, 2738 patients (37 %) received therapeutic antimicrobials for a clinical suspicion of infection, of whom 2579 (94 %) had at least two SIRS criteria and were thus diagnosed with sepsis. Table 1 presents the main characteristics of patients presenting to the ICU with a clinical diagnosis of sepsis. C-reactive protein and core temperature increased significantly with increasing likelihoods of infection, in contrast to leukocyte counts. The most frequent suspected primary sources of sepsis in the retrospective review of cases were pulmonary (community-acquired and hospital-acquired pneumonia, *n* = 1292), abdominal (peritonitis, *n* = 414), bloodstream (endocarditis, primary bloodstream, and catheter-related bloodstream, *n* = 230), urinary tract (*n* = 162), and skin or soft tissue (*n* = 118) infections. The remaining 363 patients had infections at other sites.

**Table 1:** Baseline characteristics of patients admitted with presumed sepsis

	All	Post-hoc plausibility of infection				
		None	Possible	Probable	Definite	*p* value
Number	2579 (100 %)	332 (13 %)	771 (30 %)	633 (25 %)	843 (33 %)	n/a
Demographics						
Age (years)	62 (49, 71)	62 (48, 72)	62 (51, 71)	62 (49, 71)	62 (49, 71)	0.73
Gender, male	1540 (60 %)	181 (55 %)	493 (64 %)	365 (58 %)	501 (59 %)	0.04
Race, Caucasian	2257 (88 %)	278 (84 %)	688 (89 %)	552 (87 %)	739 (88 %)	0.09
Body mass index >30 kg/m^2^	479 (19 %)	53 (16 %)	166 (22 %)	93 (15 %)	167 (20 %)	0.004
Comorbidities						
Charlson comorbidity index	3.5 (0, 9.1)	1.5 (0, 8.1)	2.5 (0.0, 9.4)	4.6 (0.0, 9.4)	4.6 (0.0, 9.7)	0.02
Cardiovascular disease^a^	605 (23 %)	81 (24 %)	204 (26 %)	142 (22 %)	178 (21 %)	0.07
Respiratory insufficiency^b^	432 (17 %)	50 (15 %)	141 (18 %)	120 (19 %)	121 (14 %)	0.05
Renal insufficiency^c^	329 (13 %)	31 (9 %)	98 (13 %)	70 (11 %)	130 (15 %)	0.02
Malignancy^d^	239 (9 %)	20 (6 %)	61 (8 %)	68 (11 %)	90 (11 %)	0.02
Immunocompromised state^e^	628 (24 %)	68 (20 %)	159 (21 %)	173 (27 %)	228 (27 %)	0.002
Diabetes mellitus	486 (19 %)	63 (19 %)	143 (19 %)	119 (19 %)	161 (19 %)	0.99
Admission characteristics						
Surgical admission	661 (26 %)	88 (27 %)	186 (24 %)	122 (19 %)	265 (31 %)	<0.001
APACHE IV score	77 (66, 100)	74 (58, 101)	75 (58, 96)	79 (61, 100)	79 (60, 101)	0.08
Core temperature	37.8 (37.0, 38.6)	37.6 (36.9, 38.4)	37.7 (37.0, 38.5)	37.9 (37.1, 38.6)	37.9 (37.1, 38.7)	<0.001
White blood cell count	14.2 (9.6, 19.8)	13.5 (9.9, 18.8)	14.6 (10.4, 19.0)	14.2 (9.6, 20.1)	14.5 (8.5, 20.5)	0.88
C-reactive protein	114 (35, 229)	36 (8, 102)	86 (19, 181)	125 (47, 234)	170 (78, 270)	<0.001
Creatinine	104 (70, 171)	101 (68, 167)	100 (69, 157)	94 (65, 158)	118 (75, 198)	<0.001
Sepsis severity at admission						<0.001
Sepsis	1076 (42 %)	175 (53 %)	380 (49 %)	238 (38 %)	283 (34 %)	
Severe sepsis	727 (28 %)	70 (21 %)	224 (29 %)	198 (31 %)	235 (28 %)	
Septic shock	776 (30 %)	87 (26 %)	167 (22 %)	197 (31 %)	325 (39 %)	
Organ failure at admission^f^						
Central nervous system	0.0 (0.0, 1.0)	0.0 (0.0, 1.0)	0.0 (0.0, 1.0)	0.0 (0.0, 1.0)	0.0 (0.0, 1.0)	0.22
Cardiovascular	3.0 (1.0, 4.0)	3.0 (1.0, 4.0)	3.0 (1.0, 4.0)	3.0 (1.0, 4.0)	3.0 (1.0, 4.0)	<0.001
Respiratory	2.5 (2.0, 3.0)	3.0 (2.0, 3.0)	3.0 (2.0, 3.0)	3.0 (2.0, 3.0)	2.0 (2.0, 3.0)	0.35
Renal	0.0 (0.0, 2.0)	0.0 (0.0, 1.0)	0.0 (0.0, 1.0)	0.0 (0.0, 1.0)	1.0 (0.0, 3.0)	<0.001
Hepatic	0.0 (0.0, 0.0)	0.0 (0.0, 0.0)	0.0 (0.0, 0.0)	0.0 (0.0, 0.0)	0.0 (0.0, 0.0)	<0.001
Coagulation	0.0 (0.0, 1.0)	0.0 (0.0, 1.0)	0.0 (0.0, 1.0)	0.0 (0.0, 1.0)	0.0 (0.0, 1.5)	<0.001
Total	7.0 (5.0, 10)	7.0 (5.0, 10)	7.0 (4.0, 9.0)	7.0 (5.0, 9.0)	8.0 (5.0, 11)	<0.001
Treatment at admission						
Mechanical ventilation	2016 (78 %)	261 (79 %)	608 (79 %)	492 (78 %)	655 (78 %)	0.93
Dialysis	263 (10 %)	39 (12 %)	67 (9 %)	54 (9 %)	103 (12 %)	0.037

Data presented as median (interquartile range) or number (%). The four infection plausibility classes were compared using the Kruskal–Wallis test or the chi-squared test

^a^Cardiovascular disease was defined as cerebrovascular disease or chronic cardiovascular insufficiency (New York Heart Association class 4), chronic congestive heart failure (ejection fraction <30 %), or peripheral vascular disease (intermittent claudication, patients with percutaneous transluminal angioplasty, or bypass for arterial insufficiency)

^b^Respiratory insufficiency was defined as chronic obstructive pulmonary disease or chronic respiratory insufficiency with functional disabilities (chronic mechanical ventilation, oxygen use at home, or severe pulmonary hypertension)

^c^Renal insufficiency was defined as chronic renal insufficiency (creatinine >177 μmol/l) or chronic dialysis

^d^Malignancy included both metastatic and hematologic malignancies

^e^Immunocompromised state was defined as having acquired immunodeficiency syndrome, the use of corticosteroids in high doses (equivalent to prednisolone of >75 mg/day for at least 1 week), current use of immunosuppressive drugs, current use of antineoplastic, drugs recent hematologic malignancy, or documented humoral or cellular deficiency

^f^Based on the Sequential Organ Failure Assessment scores

*APACHE* Acute Physiology and Chronic Health Evaluation, *n/a* not applicable

### Accuracy of infection diagnosis

**Fig. 1:**
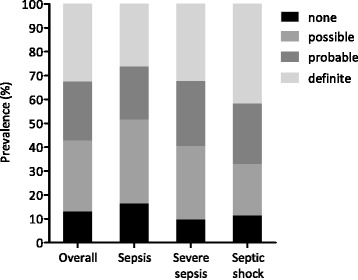
Plausibility of infection stratified by clinical severity upon presentation in patients with presumed sepsis. Comparison between the clinical diagnosis of infection at the time of ICU admission and the actual presence of infection as determined by post-hoc evaluation

**Fig. 2:**
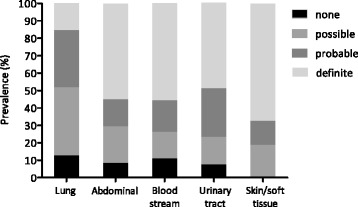
Plausibility of infection in patients with presumed sepsis upon presentation for the most frequent sites of infection. Distribution of plausibility of infection for lung infections (community-acquired pneumonia and hospital-acquired pneumonia), abdominal infections (primary and secondary peritonitis), bloodstream infections (primary bloodstream infections, catheter-related bloodstream infections, and endocarditis), urinary tract infections, and skin/soft tissue infections

Of all patients treated for sepsis, 13 % had an infection likelihood of “none” upon post-hoc analysis (Table 1). An additional 30 % had an infection likelihood of possible, whereas slightly more than half scored a higher infection likelihood (25 % probable and 33 % definite). Limiting the analysis to infections that were diagnosed within 48 hours before admission resulted in a similar distribution (*n* = 2117): 15 %, 32 %, 25 %, and 28 % were classed as none, possible, probable, and definite infections, respectively. Figure 1 shows the plausibility of infection after post-hoc analysis for the whole cohort, and stratified by sepsis severity. Although the accuracy of the infection diagnosis according to the post-hoc adjudication increased with greater sepsis severity, there was still considerable misclassification in patients with organ failure (40 % of patients classified as none or possible) or shock (34 %). Figure 2 shows the plausibility of infection after post-hoc analysis for the five most prevalent sources of infection. The proportion of definite and probable infections was largely similar in patients with different sources of infection, although the percentage of definite cases in pneumonia patients was significantly lower compared with the whole cohort (16 % vs. 33 %, *p* <0.001). Furthermore, there were no likelihoods of “none” in the cases with skin or soft tissue infection. Additional file 1 shows all (both sepsis and nonsepsis) diagnoses that were registered in patients by category of infection likelihood.

### Outcomes

**Fig. 3:**
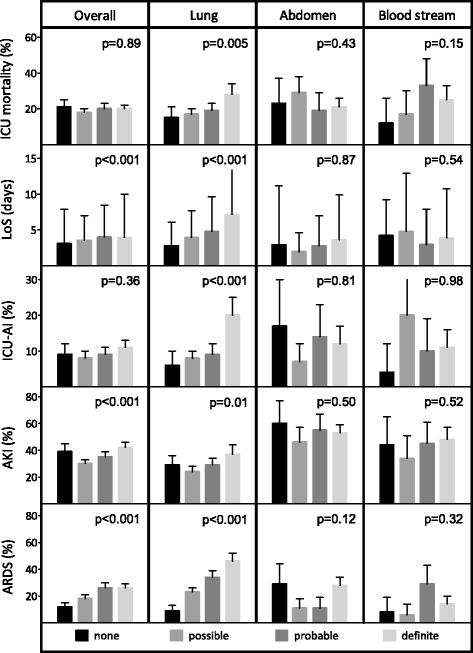
Patient outcomes for various sites of infection stratified by plausibility of infection. Data are crude associations. The length of ICU stay (*LoS*) is shown as median. ICU-acquired infections (*ICU-AI*) were defined as infections that started >48 hours after admission with a plausibility of infection of at least possible. Acute kidney injury (*AKI*) and adult respiratory distress syndrome (*ARDS*) that were present at or occurred during ICU admission were taken into account. Whiskers indicate the 95 % CI. *p* values indicate the results of the Cochran-Armitage chi-square test for trend. Urinary tract and skin/soft tissue infections are not shown because of relatively small subgroups after stratification

**Fig. 4:**
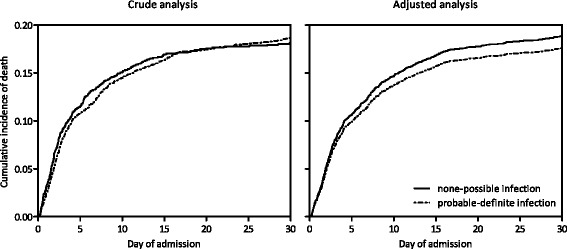
Crude and adjusted cumulative incidence functions of mortality stratified by plausibility of infection. The adjusted curve (*right*) was plotted by imputing average values of age, gender, cardiovascular disease, immunocompromised state, malignancy, diabetes mellitus, respiratory insufficiency, renal insufficiency, recent surgery, sepsis severity, site of infection, and APACHE IV score into the model

Figure 3 shows various patient outcomes in the whole population, and stratified by infection likelihood and the most prevalent presumed sources of infection. The plausibility of infection was not associated with mortality either in the entire patient population admitted with a sepsis diagnosis (21 %, 18 %, 20 %, and 20 % mortality in patients with infection likelihoods of none, possible, probable, and definite, respectively) or in any of the main subgroups of presumed infection sites except for the lungs. Figure 4 displays the cumulative incidence functions of mortality for the none–possible vs. probable–definite classes of infection plausibility. The confidence intervals for all four categories overlap, meaning that in this crude survival analysis plausibility of infection was also not associated with mortality (*p* = 0.73; crude SHR 1.05; 95 % confidence interval (CI) 0.88–1.25). In the multivariable analysis, however, a higher plausibility of infection (probable/definite) was associated with a lower mortality (SHR 0.81; 95 % CI 0.67–0.97). This means that patients with a confirmed infection diagnosis actually have a lower mortality rate than patients with an unconfirmed infection or an alternative diagnosis. Cause-specific analysis revealed that this reduction was caused by a direct effect on death (CSHR 0.73; 95 % CI 0.61–0.89), and not by the indirect effect on a longer ICU length of stay (CSHR 0.93; 95 % CI 0.85–1.02). In subgroup analyses, the mortality hazard for each hospital was similar (hospital A: SHR 0.80, 95 % CI 0.62–1.03; hospital B: SHR 0.85, 95 % CI 0.63–1.13). These estimates were similar when restricting our analysis to cases with none or definite infections only (SHR 0.75, 95 % CI 0.55–1.01). Furthermore, the prevalence of the adult respiratory distress syndrome, the prevalence of acute kidney injury, and the length of stay significantly increased with greater infection likelihoods (*p* <0.001), whereas the occurrence of ICU-acquired infections did not (*p* = 0.36) (Fig. 3). In the main subgroups of presumed infection sites, the infection plausibility was not associated with outcome parameters in this crude analysis, except for pulmonary infections.

## Discussion

We determined the accuracy of the infection diagnosis made by clinicians in the context of presumed sepsis upon admission to the ICU and found that up to 43 % of patients treated for sepsis were unlikely to have had an infection on post-hoc assessment. Although the accuracy of the infection diagnosis increased with increasing severity of disease, a considerable proportion of patients with severe sepsis and septic shock still had at most a possible infection. These results show that making an accurate infection diagnosis upon ICU admission in patients with suspected sepsis is difficult in many cases.

Our study is the first prospective comparison of sepsis diagnoses made by ICU physicians and post-hoc analyses of infection likelihoods based on strict diagnostic criteria, revealing that the true incidence of sepsis upon ICU admission is probably overestimated. Only few previous studies have specifically investigated the accuracy of infection diagnoses in patients with suspected sepsis in the ICU. A French study found that 49 % of patients were potentially unnecessarily treated for a new infection on the ICU [16]. This finding was based on the level of microbiological evidence and not on well-defined diagnostic criteria, however, making it difficult to appreciate the true percentage of patients without infection in post-hoc analysis. Another study explored the correlation of clinical certainty at the start of antimicrobial therapy with the post-hoc presence of infection [17]. The primary aim of this latter investigation focused on antimicrobial use, namely how often administration of antimicrobials for suspected infection could be justified by the presence of infection; a large proportion of patients treated with empirical antibiotics (58 of the 125; 46 %) actually had no infection according to the infectious diseases specialist in the post-hoc assessment [17].

In crude analysis, the likelihood of infection in patients treated for suspected sepsis was not associated with mortality. Since several factors that impact on ICU mortality were unequally distributed between groups, we performed multivariable survival analysis and found that a lower likelihood of infection was associated with increased mortality. In other words, patients who were initially treated for sepsis but had, in retrospect, a noninfectious diagnosis had a higher mortality rate compared with patients with an infection. This observation is probably related to variations in underlying pathology, but may also partly be due to the diagnostic delay that resulted from an incorrect working diagnosis. Furthermore, these data suggest that infection does not result in worse outcome compared with other critical conditions. It is important to note that the increasing incidences of complications such as acute kidney injury and adult respiratory distress syndrome in cases with higher plausibilities of infection are markers of a “correct” sepsis diagnosis and should not be interpreted as causal factors of the lower adjusted mortality rates in noninfectious cases.

As the discrimination between infectious and noninfectious causes of critical illness in the ICU using clinical parameters only has proved challenging, multiple studies have been performed into the value of other markers, such as host biomarkers for the diagnosis of infection [18, 19]. While some biomarkers, such as procalcitonin, may aid in limiting the duration of antibiotic therapy in ICU patients [20], at present there are no biomarkers that provide sufficient diagnostic accuracy to withhold antibiotics as initial therapy in ICU patients with suspected infection [18, 21, 22]. While biomarkers would be valuable for diagnosis in reducing antibiotic use in this patient population, our current study suggests that for stratification according to risk for an adverse outcome, the infection diagnosis itself is less important.

A limitation of this study concerns the inherently somewhat complex CDC and ISF infection definitions used for the post-hoc assessment for the presence of infection. We therefore determined the diagnostic agreement among the study team in a separate study, and concordance was found to be good [3]. In contrast to this previous study, the current process of prospective surveillance involved discussions among observers, discussions with (senior) clinicians in multidisciplinary meetings attended by critical care physicians and infection specialists, and continuous checks of data integrity. All diagnoses were therefore made after consensus. As such, our post-hoc analyses represent an “ideal” situation with availability of all diagnostic data collected after the acute event. Consequently, our study should not be interpreted as an analysis of the adequacy of clinical action in the ICU, but rather as an attempt to assess the true incidence of infection in patients admitted with suspected sepsis. In this respect it is important to note that in large surveys the rapid administration of broad-spectrum antibiotics to patients with clinically diagnosed septic shock is associated with a time-dependent increase in survival [23, 24], suggesting that the benefit of early antibiotic treatment in patients with infection is greater than the potential harm of unnecessary antimicrobial therapy in those without infection. Notably, relative to other sources of infection, only few pneumonia cases fulfilled the criteria for definite infection. This was most probably caused by a relatively strict definition for pneumonia [3]. Furthermore, any systemic use of antibiotics before admission may have influenced culture results obtained in the ICU and therefore also the recorded likelihood of infection. However, a positive culture was not necessarily needed to diagnose a probable infection, including for the most commonly observed community-acquired infection in our study (pneumonia). For patients with hospital-acquired infections, this issue was deemed less problematic since blood (and other) cultures were typically collected before the start of antibiotics. Another limitation involves the fact that this study was performed in two centers in the Netherlands and may not reflect general ICU practice. Lastly, as is true for all observational studies, we cannot rule out the possibility that unobserved confounding might have occurred in the mortality analysis. However, the adjustment methods used were identical for all subgroups.

## Conclusions

This first prospective analysis of the accuracy of the infection diagnosis in patients with suspected sepsis on ICU admission shows that the clinical diagnosis of sepsis corresponds poorly with the actual presence of infection, as defined by CDC/ISF diagnostic criteria. These results suggest that the true incidence of sepsis may have been overestimated in many studies. In fact, a substantial portion of patients being enrolled in clinical sepsis trials may in fact not have probable or definite infection, which may negatively impact the power of such trials to show benefit of certain sepsis treatments.

## Key messages

- The clinical diagnosis of sepsis on admission corresponds poorly with the presence of infection defined by strict diagnostic criteria.
- A higher likelihood of infection does not negatively impact the mortality of patients treated for sepsis.

## Additional file

Additional file 1:
**Is Table S1 presenting diagnoses in patients admitted with sepsis by infection likelihood.** Overview of all (both sepsis and nonsepsis) diagnoses that were recorded in patients upon admission by category of infection likelihood. (PDF 144 kb)

## Abbreviations

APACHE: Acute Physiology and Chronic Health Evaluation
CDC: Centers for Disease Control and Prevention
CI: Confidence interval
CSHR: Cause-specific hazard ratio
IQR: Interquartile range
ISF: International Sepsis Forum
MARS: Molecular Diagnosis and Risk Stratification of Sepsis
SHR: Subdistribution hazard ratio
SIRS: Systemic inflammatory response syndrome
