# Supplementary material for: Likelihood of infection in patients with presumed sepsis at the time of intensive care unit admission: a cohort study
Source: Crit Care. 2015 Sep 7;19(1):319. doi: 10.1186/s13054-015-1035-1 (PMC4562354; doi:10.1186/s13054-015-1035-1)
Supplement: Additional file 1: — Is Table S1 presenting diagnoses in patients admitted with sepsis by infection likelihood. Overview of all (both sepsis and nonsepsis) diagnoses that were recorded in patients upon admission by category of infection likelihood. (PDF 144 kb) [file 13054_2015_1035_MOESM1_ESM.pdf]

ONLINE DATA SUPPLEMENT

Presence of infection in patients with presumed sepsis at the time of intensive care unit admission: a cohort study

Peter M.C. Klein Klouwenberg, Olaf L. Cremer, Lonneke A. van Vught, David S.Y. Ong, Jos F. Frencken, Marcus J. Schultz, Marc J. Bonten, Tom van der Poll

Table S1 Diagnoses in patients admitted with sepsis by infection likelihood

None	332	Possible	771	Probable	633	Definite	843
**Cardiovascular**	**117**	**Cardiovascular**	**203**	**Infectious**	**253**	**Infectious**	**310**
Cardiac arrest	26	Cardiac arrest	79	Pneumonia	161	Sepsis	112
Congestive heart failure	13	Congestive heart failure	34	Sepsis	65	Pneumonia	93
Cardiovascular surgery	6	CABG	13	Endocarditis	8	Endocarditis	22
Rhythm disturbance	5	Cardiovascular surgery	10	Surgery for infection	6	Surgery for infection	17
Cardiomyopathy	5	Cardiogenic shock	9	Soft tissue infection	4	Soft tissue infection	17
**Neurologic**	**65**	**Infectious**	**156**	**Cardiovascular**	**191**	**Gastrointestinal**	**210**
Subarachnoid hemorrhage	16	Pneumonia	92	Cardiac arrest	29	Perforation	49
Seizures	7	Sepsis	43	Congestive heart failure	12	Compl. of previous bleeding	34
Coma	5	Renal infection	7	Cardiomyopathy	5	Upper GI bleeding	11
Intracranial hemorrhage	4	Infection/abscess	4	Cardiogenic shock	4	Surgery	10
Overdose	3	Soft tissue infection	3	Hemorrhage	4	Vascular ischemia	7
**Respiratory**	**50**	**Neurologic**	**119**	**Gastrointestinal**	**82**	**Cardiovascular**	**90**
Emphysema	12	Subarachnoid hemorrhage	26	Perforation	22	Congestive heart failure	14
Pleural effusions	5	Seizures	16	Compl. of previous surgery	4	Cardiac arrest	11
Astma	4	Coma	13	Hemorrhage	4	Aortic valve replacement	5
ARDS	3	Stroke	12	Surgery	4	Cardiovascular surgery	4
Atelectasis	3	Subdural hematoma	5	Vascular ischemia	3	Abdominal aortic aneurysm	4
**Infectious**	**39**	**Respiratory**	**118**	**Neurologic**	**81**	**Respiratory**	**81**
Pneumonia	22	Emphysema	16	Subarachnoid hemorrhage	15	Pleural effusions	11
Sepsis	11	Atelectasis	13	Stroke	9	ARDS	11
Endocarditis	4	Airway obstruction	13	Seizures	7	Atelectasis	10
Renal infection	2	Pleural effusions	11	Intracranial hemorrhage	4	Airway obstruction	8
		Pulmonary embolus	8	Subdural hematoma	4	Emphysema	7
**Gastrointestinal**	**38**	**Gastrointestinal**	**100**	**Respiratory**	**72**	**Neurologic**	**35**
Hemorrhage	9	Perforation	17	ARDS	11	Coma	4
Compl. of previous surgery	4	Vascular ischemia	9	Emphysema	8	Subarachnoid hemorrhage	4
Perforation	3	Compl. of previous surgery	7	Atelectasis	7	Intracranial hemorrhage	4
GI surgery	3	Obstruction	7	Pleural effusions	6	Overdose	3
Obstruction	2	Hemorrhage	4	Airway obstruction	6	Subdural hematoma	3

The top five of diagnoses per organ system are shown. Not shown are less frequently occurring metabolic, hematologic and genito-urinary organ systems, trauma, and transplantation. Abbreviations: ARDS adult respiratory distress syndrome; CABG coronary artery bypass grafting; Compl. Complications; GI gastro-intestinal.
